# Twelve-hour normothermic liver perfusion in a rat model: characterization of the changes in the ex-situ bio-molecular phenotype and metabolism

**DOI:** 10.1038/s41598-024-56433-3

**Published:** 2024-03-13

**Authors:** Daniele Dondossola, Caterina Lonati, Michele Battistin, Luigi Vivona, Alberto Zanella, Marco Maggioni, Vaira Valentina, Laimdota Zizmare, Christoph Trautwein, Andrea Schlegel, Stefano Gatti

**Affiliations:** 1 General and Liver Transplant Surgery Unit, Fondazione IRCCS Ca’ Granda Ospedale Maggiore Policlinico, Via Francesco Sforza 35, 20100 Milan, Italy; 2 Department of Pathophysiology and Transplantation, University of Milan, Via Francesco Sforza 35, 20100 Milan, Italy; 3 Center for Preclinical Research, Fondazione IRCCS Ca’ Granda Ospedale Maggiore Policlinico, Via Pace 9, 20100 Milan, Italy; 4 Department of Anesthesia, Critical Care and Emergency, Fondazione IRCCS Ca’ Granda - Ospedale Maggiore Policlinico, Milan, Italy; 5 Division of Pathology, Fondazione IRCCS Ca’ Granda Ospedale Maggiore Policlinico, Milan, Italy; 6 Werner Siemens Imaging Center, Department of Preclinical Imaging and Radiopharmacy, University Hospital Tübingen, Eberhard Karls University of Tübingen, Röntgenweg 13, 72076 Tübingen, Germany; 7 Transplantation Center, Digestive Disease and Surgery Institute and Department of Immunology, Lerner Research Institute, Cleveland Clinic, Cleveland, OH USA

**Keywords:** Liver, Preclinical research

## Abstract

The partial understanding of the biological events that occur during normothermic machine perfusion (NMP) and particularly during prolonged perfusion might hinder its deployment in clinical transplantation. The aim of our study was to implement a rat model of prolonged NMP to characterize the bio-molecular phenotype and metabolism of the perfused organs. Livers (n = 5/group) were procured and underwent 4 h (NMP4h) or 12 h (NMP12h) NMP, respectively, using a perfusion fluid supplemented with an acellular oxygen carrier. Organs that were not exposed to any procedure served as controls (Native). All perfused organs met clinically derived viability criteria at the end of NMP. Factors related to stress-response and survival were increased after prolonged perfusion. No signs of oxidative damage were detected in both NMP groups. Evaluation of metabolite profiles showed preserved mitochondrial function, activation of Cori cycle, induction of lipolysis, acetogenesis and ketogenesis in livers exposed to 12 h-NMP. Increased concentrations of metabolites involved in glycogen synthesis, glucuronidation, bile acid conjugation, and antioxidant response were likewise observed. In conclusion, our NMP12h model was able to sustain liver viability and function, thereby deeply changing cell homeostasis to maintain a newly developed equilibrium. Our findings provide valuable information for the implementation of optimized protocols for prolonged NMP.

## Introduction

Machine perfusion (MP) is a clinically applied technology aimed to evaluate, preserve and improve organ quality before transplantation, but its full potential is still under investigation. Clinical trials based on hypothermic oxygenated MP (HOPE) show a significant reduction of complications after liver transplantation (LT)^1^. An improved energy pool and succinate metabolism are the main protective mechanisms whereby HOPE-treated organs showed reduced reperfusion injury after transplantation^2,3^. In contrast, the biological processes elicited by normothermic MP (NMP) are still poorly described^4^.

There is a growing clinical interest in prolonging preservation time by MP and in recent years an increasing number of studies has been focused on prolonged reperfusion of human livers^5–8^. In addition to the tremendous logistic advantages^6^, an extension of perfusion time could provide an adequate time-window for more efficient marginal organ recovery. In fact, the efficacy of any reconditioning strategy, based on either pharmacological interventions^9^ or endogenous resolution/repair mechanisms^10^, depends on an adequate perfusion time. Prolonged perfusion appears particularly promising in the context of NMP, where the physiological temperature allows full activation of the biological processes required to boost repair/regeneration^10–12^ and to modulate the liver immunological function^13^. However, extended MP under normothermic conditions requires many crucial issues to be addressed, including adequate supply of fresh nutritional factors to hepatocytes, optimization of perfusion parameters, and identification of effective strategies for optimal oxygen delivery. Set up of a safe procedure is likewise essential to counterbalance the potential negative effects exerted by the mild ischemia/reperfusion (IR) hit inherent in the NMP procedure itself^14,15^, which could significantly impact the bio-molecular and metabolic liver phenotype in the later phases of reperfusion.

In this scenario, the design of preclinical protocols for prolonged MP could open the pathway to the use of preclinical NMP as broad research platform, while improving the general understanding of the possible profound changes in hepatic homeostasis occurring during the extended procedure^16^. We therefore aimed to develop a stable and reproducible protocol for prolonged NMP of rat livers and then investigated the macroscopic and bio-molecular changes, either detrimental or protective, that occur throughout the procedure and how they evolve during a prolonged perfusion. The rationale behind the selection of rodent model relies on its several advantages compared to large animals, including a more rapid recovery of hepatic homeostasis and function, less intra-experimental group variability, availability of genetically modified animals, and greater selection of specific laboratory reagents. To this aim, we modified our previously described protocol of rodent liver 4 h-NMP^17,18^, based on an artificial non-cellular Oxygen Carrier (OxC), by extending the perfusion time to 12 h. This timeframe was selected based on a previous study focused on gene expression profiling of in-vivo resected livers^19^, where we showed that multiple endogenous adaptation processes take place in liver cells during the 12 h immediately after exposure to stress stimuli.

## Results

### Hemodynamic parameter’s during NMP

Portal resistance decreased during the initial rewarming phase. Afterwards, during the “normothermic perfusion phase” and without pharmacological interventions, no vasoconstriction occurred, and the portal resistance remained stable with comparable values in both groups (p = 0.473) (Supplementary Fig. 1).

### Oxygen consumption and release of markers of liver viability during NMP

**Figure 1:**
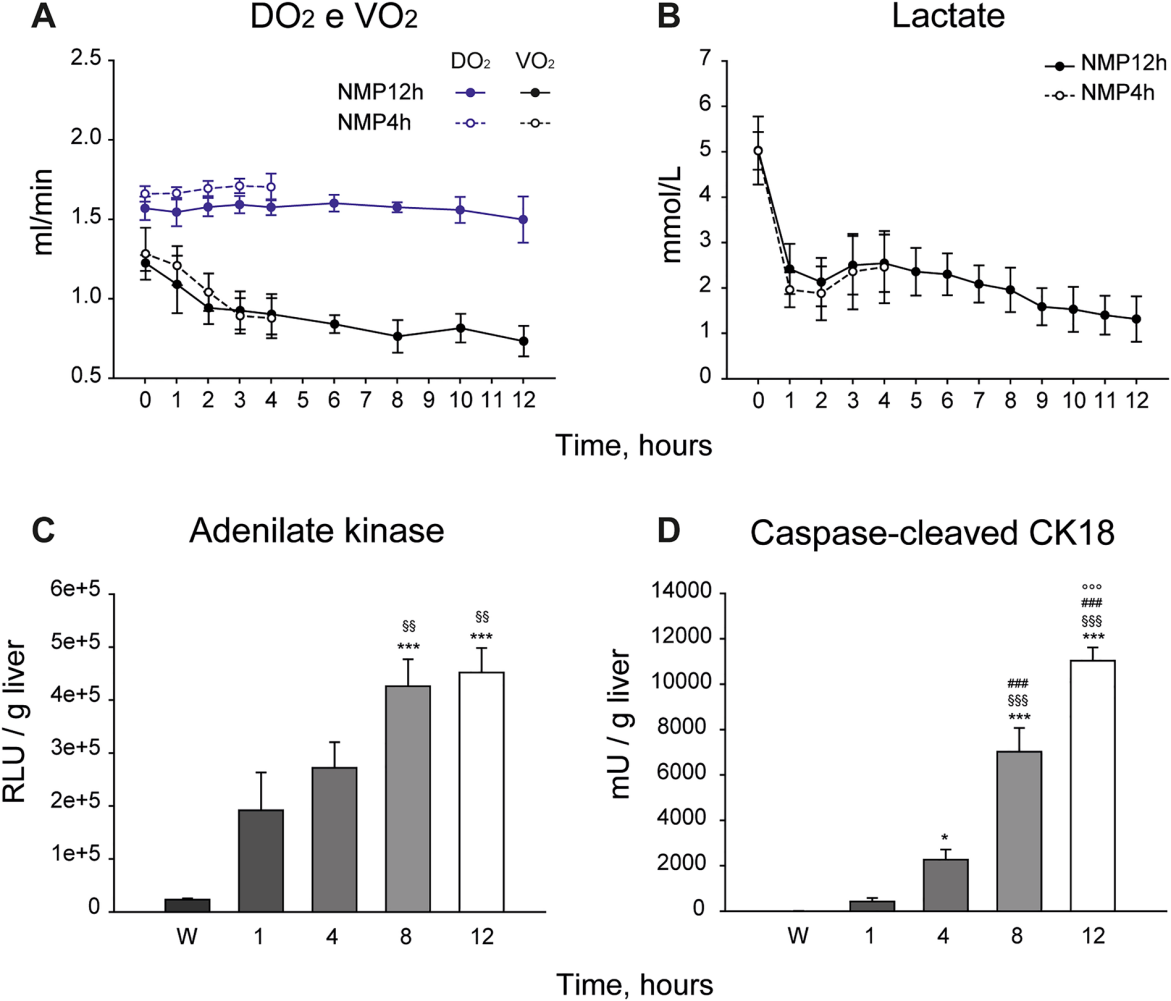
Oxygen consumption and release of markers of liver viability during the prolonged NMP procedure. (**A**) Oxygen delivery (DO_2_) was stable throughout the perfusion procedure (p = 0.873), while oxygen consumption (VO_2_), after an intial decrease from 0 to 2 h of perfusion, remained then stable; Two-way repeated measures ANOVA, Tukey’s post hoc test. (**B**) Lactate concentration decreased over time in both study groups (p < 0.001 vs time; p = 0.683 vs 4 h), despite 7.99 mmol/h lactate were continuously infused; Two-way repeated measures ANOVA, Tukey’s post hoc test. (**C**) and (**D**) Release of biomarkers of liver cell viability during NMP. Caspase cleaved cytokeratin 18 (CK18) and adenylate kinase (AK) were assessed in perfusate samples. One-way repeated measures ANOVA, Tukey’s post hoc test; p value vs Wash-out (W) ***p < 0.001, *p < 0.05; p value vs 1 h ^§§§^p < 0.001, ^§§^p < 0.01; p value vs 4 h ^###^p < 0.001; p value vs 8 h ^°°°^p < 0.001.

Thanks to the continuous substitution of perfusion fluid, Oxyglobin and Met-Oxyglobin concentration remained stable during the whole perfusion and did not differ in the two study groups (Met-Oxyglobin: NMP4h 30 ± 4% vs NMP12h 29 ± 4%, p = 0.459; Supplementary Table 1). The DO_2_ remained stable during the “normothermic” phase despite Met-Oxyglobin formation (Fig. 1A). Lactate uptake ratio was NMP4h 0.51 ± 0.12 and NMP12h 0.75 ± 0.01 (p = 0.010; lactate concentrations are shown in Fig. 1B). Glucose uptake ratio was 0.21 ± 0.11 in NMP 4 h and 0.45 ± 0.01 in NMP 12 h (p = 0.008; Supplementary Fig. 2). None of the evaluated variables showed any statistically significant differences between the NMP4h and NMP12h groups.

Markers of hepatocellular necrosis during NMP are shown in Supplementary Fig. 3. The perfusate concentration of the cytolysis index adenylate kinase (AK) and the apoptotic index caspase-cleaved cytokeratin 18 (CK18) increased over time (Fig. 1C,D). Of note, while AK release did not change from 8 to 12 h of perfusion, CK18 concentration showed a steeper increase between 4 and 8 h of perfusion. Evaluation of FMN release in the perfusate disclosed a rise of this factor at 8 and 12 h of NMP (Supplementary Fig. 4).

### Evaluation of hepatic cell viability and liver tissue Integrity

Liver weight at the end of perfusion was 14.2 ± 1.9 g in NMP4h and 15.1 ± 1.9 g in NMP12h. During NMP the liver weight decreased by 0.9 ± 0.5 g in NMP4h and 0.6 ± 0.5 g in NMP12h (p = 0.043).

**Figure 2:**
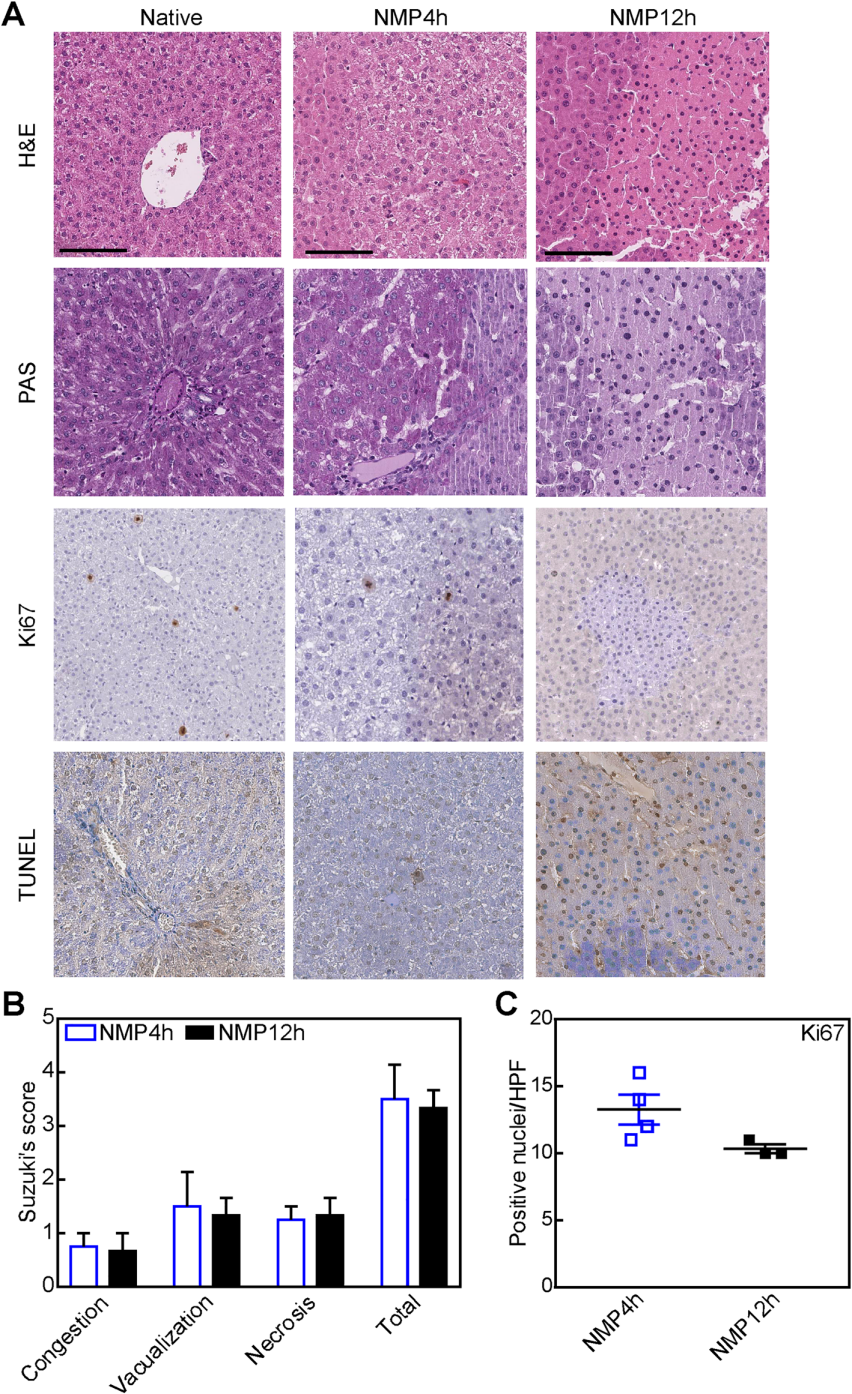
Histopathology analyses of the liver tissue at the end of prolonged NMP. (**A**) Representative of Hematoxylin and eosin (H&E—magnification 500x), Ki67 antigen (magnification 200x), PAS (magnification 50x) and TUNEL (magnification 50×) stain. (**B**) Graphical representation of Suzuki’s histological score and (**C**) Ki67 positive cells. Data are expressed as mean ± SEM.

**Figure 3:**
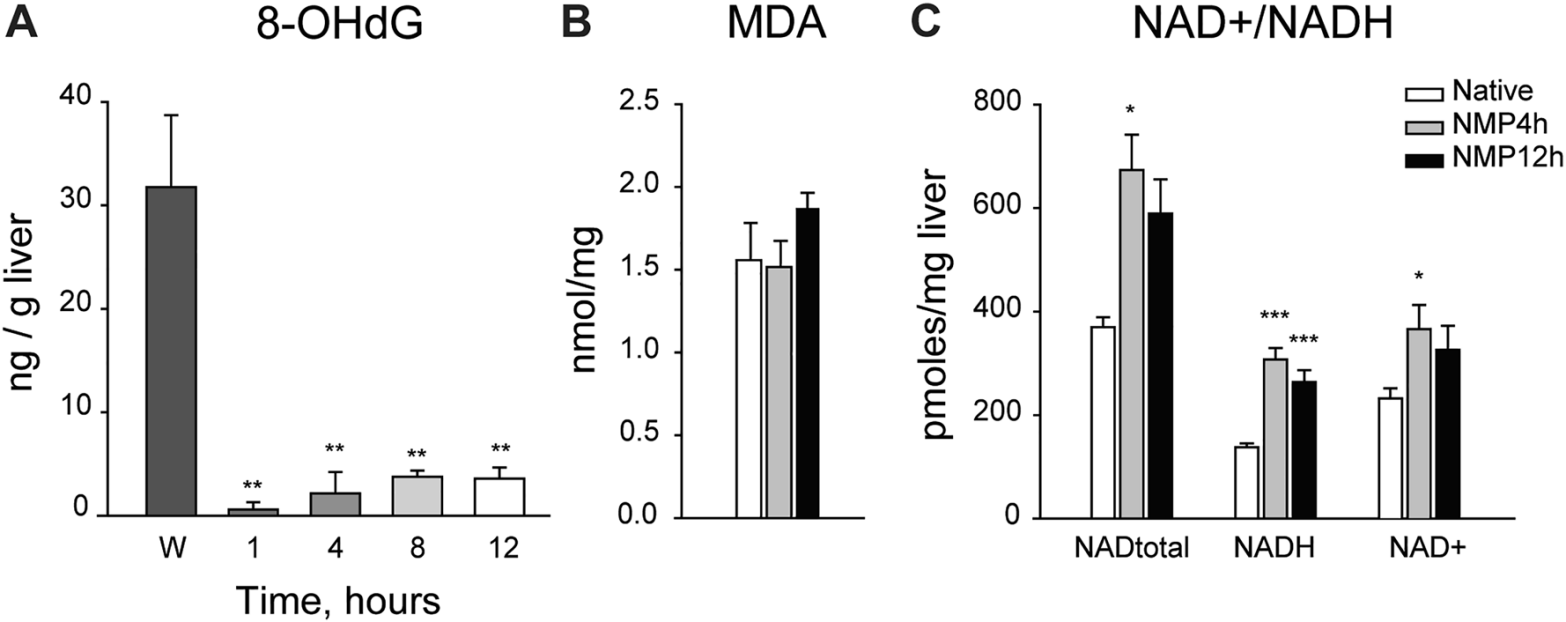
Markers of oxidative stress during prolonged NMP. (**A**) An increased concentration of 8-hydroxydeoxyguanosine (8-OHdG) was observed in wash-out (W) samples, reflecting the oxidative stress suffered during liver procurement and cold storage; One-way repeated measures ANOVA, Tukey’s post hoc test; p value vs Wash-out (W): **p < 0.01. (**B**) Malondialdehyde (MDA) was measured to assess oxidative stress-dependent lipid peroxidation in snap-frozen liver biopsies; One-way ANOVA. (**C**) NAD+/NADH content in liver biopsies; One-way ANOVA, Tukey’s post hoc test; p value vs native: *p < 0.05; ***p < 0.001.

As demonstrated by liver histology, the organ structure and morphology were preserved after 4 h and 12 h of NMP. Compared to NMP4h group, liver tissue showed a decreased Ki67% expression after 12 h of NMP, while no changes in Suzuki’s score were observed (Fig. 2). Conversely, TUNEL staining did not differ between the two study groups (Fig. 2). PAS staining showed focal area of PAS consumption (Fig. 3; Supplementary Fig. 5).

### Bile analyses

Bile production was maintained during the “normothermic” phase. A total of 3.0 ± 1.7 g and 11.9 ± 1.8 g was produced after 4 and 12 h of perfusion, respectively (Table 1). Consistently, the bile output was 1.05 ± 0.28 g/h in the NMP4h group and 1.03 ± 0.30 g/h in NMP12h (p = 0.636). Bile and perfusate pH showed a positive linear association (r = 0.645; p = 0.008). Bile composition and viability parameters are shown in Table 1.

**Table 1:** Bile characteristics and bile derived parameters in the two study groups. T-test.

	NMP4h	NMP12h	p value
pH	7.17 ± 0.04	7.22 ± 0.07	0.057
HCO_3_ (mmol/L)	13 ± 2	14 ± 2	0.583
Glucose (mg/dL)	72 ± 33	53 ± 17	0.053
Lactate (mmol/L)	2.7 ± 0.8	1.7 ± 0.6	0.037
pH bile—perfusate	0.18 ± 0.03	0.19 ± 0.05	0.185
ΔHCO_3_ bile-perfusate (mmol/L)	3.02 ± 0.98	3.38 ± 1.31	0.552
ΔGlucose bile-perfusate (mg/dL)	− 173 ± 40	− 141 ± 33	0.072
Glucose bile/perfusate	0.30 ± 0.16	0.13 ± 0.8	0.047

Δ delta.

### Release of biomarkers relevant to cell injury, extracellular matrix fragments, and acute phase response

Immunofluorescent assays indicated no significant difference in the release of markers of hepatocyte toxicity/damage and of LSEC activation during 4 h and 12 h of NMP (Table 2). To further evaluate endothelial dysfunction, extracellular matrix and glycocalyx shedding was assessed through perfusate measurement of Glycosaminoglycan (GAG) fragments—i.e., heparan sulfate, chondroitin sulfate, dermatan sulfate, keratan sulfate, and hyaluronan. Compared to wash-out, GAGs were significantly higher at all the evaluated time-points (p < 0.001), but they did not increase from 1 to 12 h of NMP (Supplementary Fig. 6). Conversely, an increase in concentrations of acute phase response proteins alpha-1 acid glycoprotein (AGP) and alpha 2 macroglobulin (A2M) and of the protective factor adiponectin was observed during the NMP procedure.

**Table 2:**
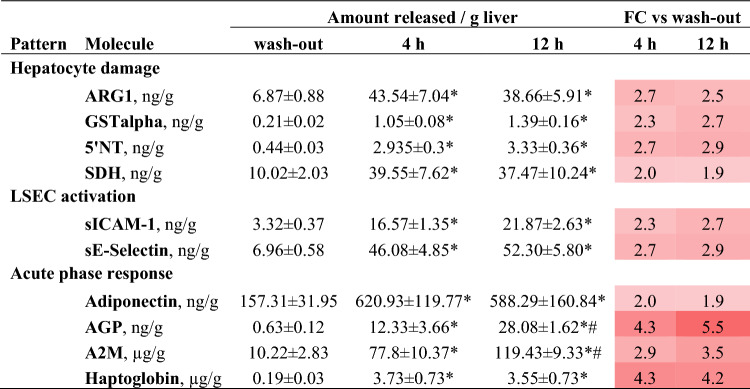
Release of biomarkers in the perfusate over the 12 h-NMP procedure. Biomarker concentration was determined by means of immunofluorescence assays.

To adjust for liver size, all the measured variables are shown as total release per gram of liver graft. Mean ± SEM (n = 5 each group), One-way repeated measures ANOVA: p < 0.001 for all the measured variables; Tukey’s post hoc test; p value vs wash-out: *p < 0.05; p value vs 4 h: ^#^p < 0.05.

5′-NT/CD73, 5′-Nucleotidase; A2M, alpha 2 Macroglobulin; AGP, Alpha-1 Acid Glycoprotein; ARG1, arginase 1; FC, Fold change; GSTα, α-glutathione S-transferase, LSEC, liver sinusoidal endothelial cells; SDH, sorbitol dehydrogenase.

### Markers of oxidative stress

In order to investigate oxidative damage, 8-hydroxy-2'-deoxyguanosine (8-OHdG) was assessed in perfusate. There was a substantial release of 8-OHdG during the wash-out phase, but then its concentration remained stable over NMP (Fig. 3A). The effect of oxidative stress on liver tissue was assessed through the evaluation of malondialdehyde (MDA) content, as a marker of lipid peroxidation, and by assessing the NAD^+^/NADH ratio. Total MDA was comparable between native livers and those subjected to both standard and prolonged NMP (Fig. 3B). The NAD^+^/NADH ratio was significantly different in ex-situ perfused livers compared to the native group (0.82 vs 1.42, p = 0.027). This change in the oxidant-antioxidant balance is mostly explained by higher NADH content in organs subjected NMP relative to native (Fig. 3C). The release of this metabolite was likewise measured in the perfusate in which it dropped after an increase up to 8 h of perfusion (Supplementary Fig. 7).

### Hepatic cell metabolism and energy charge in perfused livers

**Figure 4:**
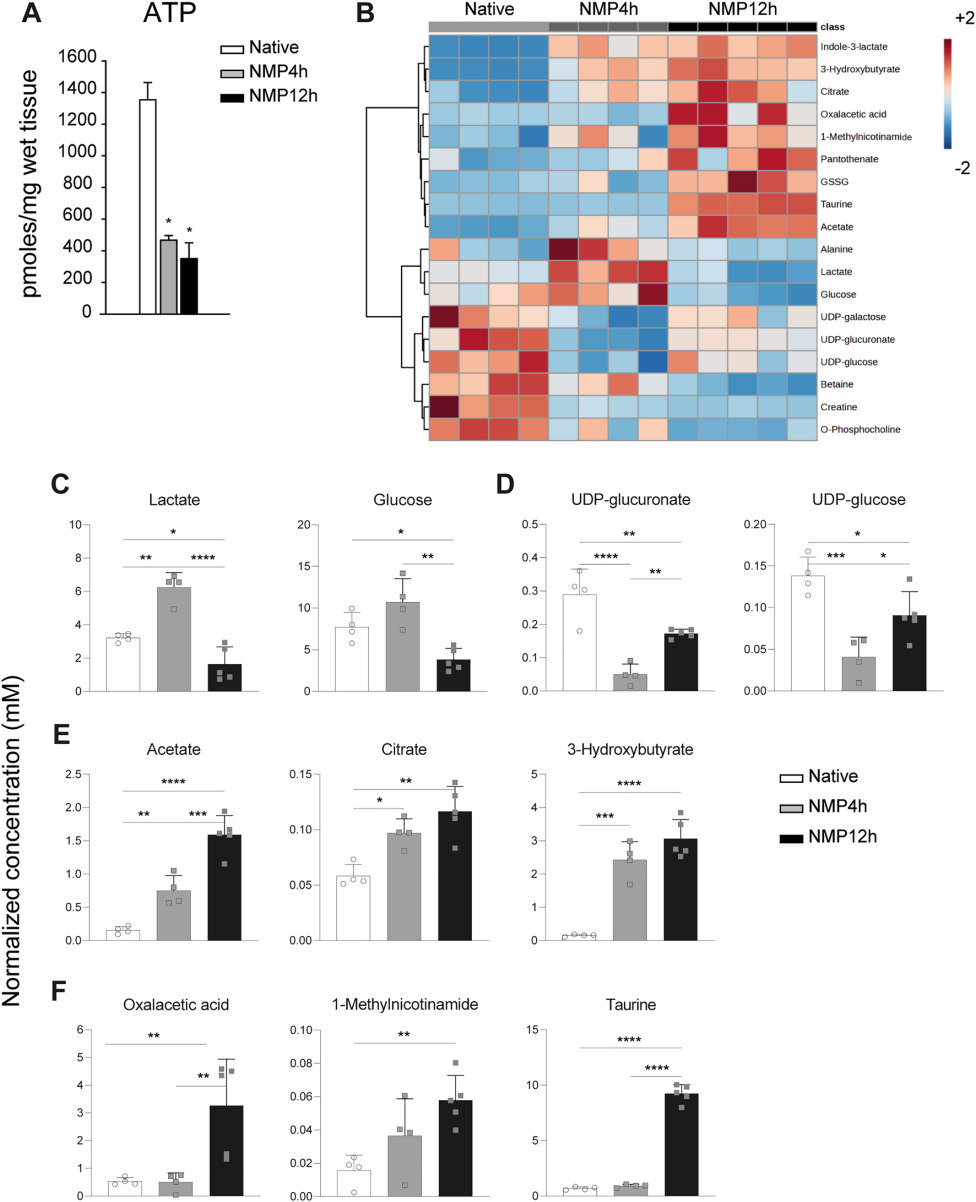
Cell metabolism and energy charge in livers subjected to prolonged NMP. (**A**) ATP content in liver tissue biopsies. Bars denote mean ± SEM; One-way ANOVA, Tukey’s post hoc test; p value vs native: *p < 0.05 NMR spectroscopy-based metabolomic analysis was performed to assess the concentration of specific metabolites in liver homogenates. (**B**) Top 18 significant metabolite heatmap illustrating individual sample metabolite concentration variation; Ward clustering algorithm, auto scaled concentration values between -2 and 2 (red—high, blue—low). (**C**) Energy metabolism-related metabolites lactate and glucose showed a reduced concentration in livers exposed to prolonged NMP. (**D**) UPD-glucuronate and UDP-glucose content was lower in perfused organs compared to native livers. (**E**) Acetate, citrate, and 3-hydroxybutyrate were induced by both short-term and prolonged NMP. (**F**) Oxalacetic acid, 1-methylnicotinamide, and taurine displayed increased concentrations in the NMP12h group. Bar plots illustrate mean ± SD; One-way ANOVA, Fisher’s post hoc test. White bar, white circles, native group; light grey bar, grey squares, NMP4h; black bar, grey squares, NMP12h. ****p < 0.0001, ***p < 0.001, **p < 0.01, *p < 0.05.

Evaluation of ATP in liver homogenates revealed that hepatic energetic pool progressively decreased over the NMP procedure (Fig. 4A).

**Figure 5:**
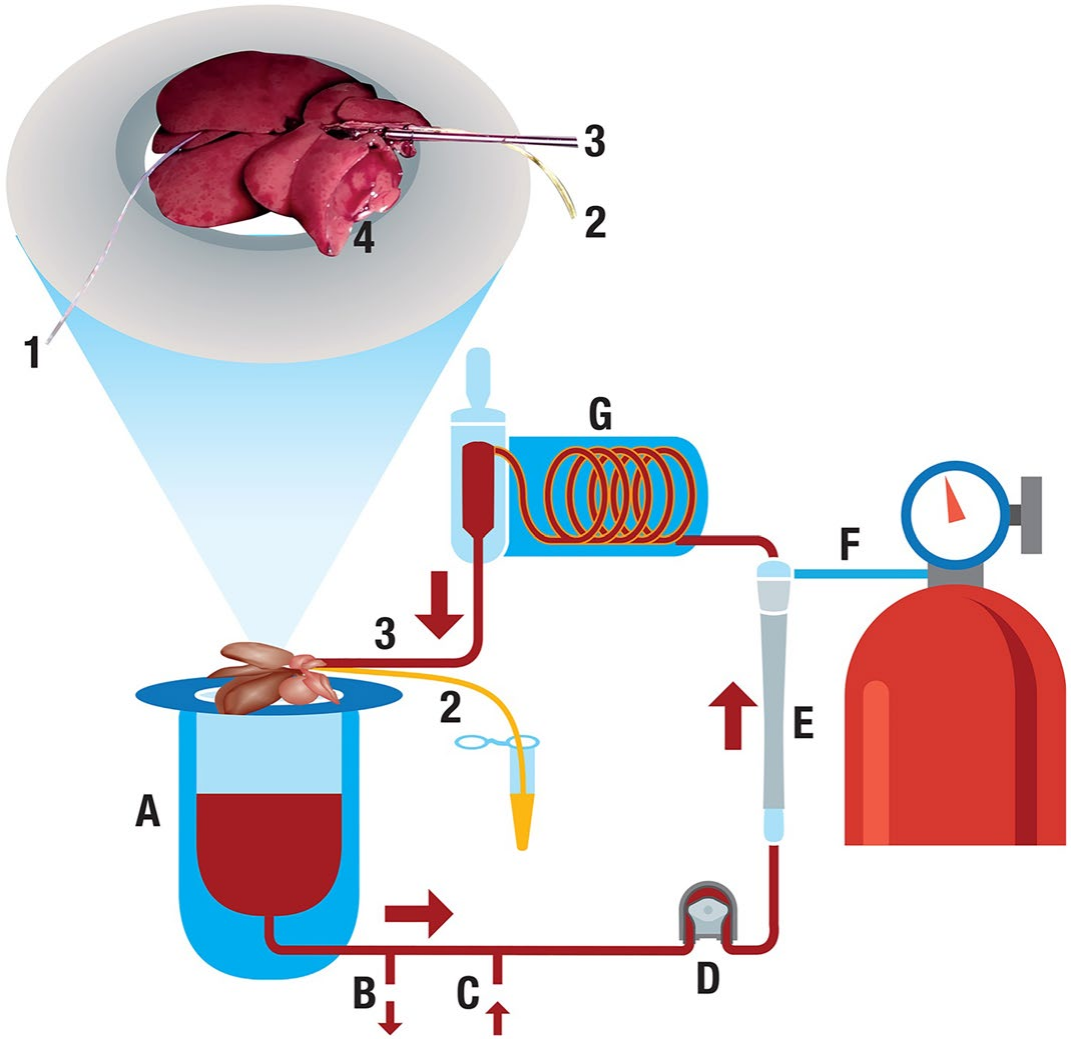
Schematic representation of the normothermic machine perfusion circuit. 1, thermal probe; 2, Bile duct tube; 3, portal vein cannula (portal flow 30 mL/min); 4, cava vein for perfusate drainage; (**A**) reservoir; (**B**) suction line used to remove perfusate; (**C**) inflow line to add fresh perfusate to the circuit (20 mL/h); (**D**) pump; (**E**) membrane oxygenator; (**F**) gas flow (50% O_2_–5% CO_2_ at a flow of 200 mL/min); G, bubble trap and heating coil with a stopcock for perfusate sampling.

The concentration of selected metabolite was assessed in tissue biopsies to investigate changes in liver cell metabolism. Of interest, livers exposed to either short-term or prolonged NMP were associated with specific metabolite signatures, which were clearly different from the profile observed in Native group (Fig. 4B, Supplementary Fig. 8). More specifically, lower lactate and glucose concentrations were observed after 12 h-NMP compared to both NMP4h and native groups (Fig. 4C). In addition, the perfusion procedure, irrespective of its duration, resulted in lower tissue content of glucoronate, betaine, creatine, and O-phosphocholine (Supplementary Fig. 8). Of note, the concentration of the UDP-sugars was reduced during 4 h-NMP, while tended to restored to baseline levels after prolonged procedure (Fig. 4B–D). Conversely, increased tissue content of acetate, citrate, and 3-hydroxybutyrate was detected in both NMP4h and NMP12h groups compared to native livers (Fig. 4E). Acetoin, succinate, cytidine, nicotinamide ribotide, indole-3-lactate, and methylguanidine showed a similar pattern of modulation (Fig. 4B, Supplementary Fig. 8). Finally, among metabolites specifically induced after prolonged perfusion, there were oxalacetic acid (Fig. 4F), 1-methylnicotinamide (Fig. 4F), taurine (Fig. 4F), glutathione disulfide (GSSG) (Fig. 4B, Supplementary Fig. 8), hypotaurine (Fig. 4B, Supplementary Fig. 8), and panthothenate (Fig. 5B, Supplementary Fig. 8).

## Discussion

This study shows for the first time feasibility to prolong NMP of rat livers to 12 h. The present optimized perfusion protocol enables to preserve liver function over an extended interval. Of note, our findings suggest that 12 h perfusion represent an adequate time-window to activate adaptive mechanisms to IRI and liver injury.

Liver MP is routinely used in the clinical setting to improve post-LT results. Despite initial encouraging results, the clinical application of NMP is presently debated due to the incomplete understanding of its effectiveness and of the mechanisms elicited. Therefore, the comprehension of NMP-related effects could therefore add further value to this technology as a platform for liver investigation and, in this regard, small animal models could be particularly helpful. However, standardized and reproducible small animal NMP models are still poorly described.

Proper oxygen availability to parenchymal cells is crucial during ex situ perfusion to support mitochondrial functional recovery after ischemia^20^ and liver metabolic activation^17,18,20,21^. Indeed, our previous metabolic investigation of rat livers ex situ-perfused for 4 h using a perfusion fluid supplemented with the non-cellular hemoglobin Oxyglobin®, disclosed improved lactate clearance, higher ATP tissue content, and lower succinate tissue and perfusate concentration in livers subjected to OxC-based NMP compared to those perfused in the absence of OxC. The present study shows that this compound, similar to the one used in clinical setting^22,23^, can effectively deliver oxygen and consequently support liver cell metabolism during prolonged NMP, as attested by analysis of DO2, VO2, and pO2. Moreover, the use of an artificial OxC enabled to fulfill the 3R principles^24,25^ of preclinical research without adding possible confounding factors to biological analyses. Indeed, the utilization of human red blood cells could have introduced significant bias due to cross-species immunological reactions and cell entrapment in different organs, which in turn can lead to impaired microcirculation^26^.

Met-Oxyglobin formation and vasoconstriction were the main concerns related to the use of this agent^27,28^. To maintain a steady Met-Oxyglobin concentration, a continuous reinfusion system to the circuit was successfully used and oxygen delivery was not affected. In addition, we did not observe increased portal vein resistances that were actually lower than elsewhere reported^18,29^. Ultimately, our perfusion parameters, maintained for prolonged timeframe, possibly serve as reference values for future “healthy control” groups to assess and compare the quality of different perfusion protocol and to improve NMP procedures.

Basic metabolic parameters, bile composition and histological analysis are currently used to assess graft viability during NMP^4,30–32^. In the present study, lactate clearance was preserved, and histology showed only slight necrosis, consistent with other results^33^. It should be noted that, in NMP groups, due to fresh perfusion fluid supplementation, lactates are continuously infused and cleared by the functioning liver from both the perfusate and liver tissue. While endogenous lactate metabolism can be sustained by a very small number of vital hepatocytes^34^, an exogenous lactate challenge would imply the upregulation of cellular enzymes involved in lactate metabolism, a potential future key test to predict effective liver function after transplantation^35,36^. In addition, the direct association between bile and perfusate pH/HCO_3_ shows that perfusate acid–base balance directly affect some of the clinically used parameters to assess cholangiocyte viability during NMP. These data suggest a cautious approach with the use of biliary chemistry during NMP as main parameter to accept or decline livers for transplantation^17^.

To obtain a deeper understanding on hepatic cell integrity and viability during prolonged perfusion, we assessed the release of biomarkers relevant to LSEC activation, glycocalyx shedding, and oxidative stress. No significant increase in markers of liver injury/toxicity (ARG1, GSTα, SDH, 5′-NT) and in GAG fragments was observed in perfusates collected after 12 h-perfusion compared to samples withdrawn at 4 h. The release of E-Selectin and sICAM-1 was likewise similar over the NMP procedure. Finally, perfusate concentration of oxidized derivative of guanosine nucleoside was steady from 1 to 12 h perfusion, indicating no further oxidative stress occurred during prolonged NMP.

Liver synthetic function and energetic status were likewise investigated over the 12-h procedure. Perfusate analysis disclosed enhanced production of factors involved in liver acute phase response and of the pleiotropic protective factor Adiponectin. This remarkable finding not only demonstrates preserved synthetic function in livers undergoing prolonged NMP, but also indicated that the duration of our protocol is adequate to allow activation of endogenous pathways related to stress response. Activation of these multiple cellular adaptation mechanisms eventually leads to the lower energetic charge observed in NMP12h. Indeed, together with the detrimental effects exerted by IRI, the depletion of energy pool could be secondary to the upregulation of the several molecular and metabolic pathways needed to cope with ischemic injury and reperfusion stress. Interestingly, in a rat model of lung ex-vivo perfusion, we previously showed that tissue adaptation to ex-situ perfusion occurs through activation of ATP-consuming biological events^14,37–39^. In addition, our metabolomic analyses highlights the activation of energy consuming processes over the 12 h-NMP, such as glycogen synthesis, glucuronidation, and bile acid conjugation^40,41^.

This being said, the lower glucose and lactate content, together with the higher oxalacetic acid tissue concentration and reduced NADH perfusate concentrations, observed after 12-h perfusion, collectively indicate that our protocol allowed hepatic cell recovery from the post-ischemic metabolic adaptations, with Cori cycle and Kreb’s cycle activation. Such interesting results not only demonstrate a preserved mitochondrial integrity in healthy livers subjected to prolonged perfusion in our system, but also confirm a key role of mitochondrial metabolism^42^ in both early and late phases of ex situ perfusion, in line with our previously published data^17^.

Recently, distinctive liver metabolic pathways were identified during clinical ex-situ perfusion^43^. Interestingly, our data support these findings and add some insights into these metabolic mechanisms. Indeed, the higher tissue content of acetate and 3-hydroxybutirate observed in ex situ-perfused livers reflects activation of lipolysis, acetogenesis^44^, and ketogenesis^45^. Moreover, the NMP12h group showed a marked increase in GSSG. Since no oxidative damage was detected in cell components, this finding suggests that the endogenous anti-oxidant factors successfully counteracted the production of reactive oxygen species. However, these observations collectively point out the need to develop tailored strategies for exogenous supplementation during the NMP procedure^16,43^. This appears a key aspect as the availability of metabolic substrates is essential to trigger liver regeneration and inflammation resolution^46^, otherwise the same adaptive mechanisms could lead to cell death and possible liver failure.

Finally, compared with native livers, an increase in NADH content and NAD^+^/NADH ratio was revealed in organs subjected to 12 h of perfusion. These findings could depend on different hypothesis: activation of autophagy (essential for hepatocyte survival after nutrient deprivation and hypoxia)^47–49^, or on incomplete functional recovery of respiratory complex I NADH-dehydrogenase, a phenomenon already described during NMP^50,51^. A third hypothesis is supported by the higher concentration of 1-methylnicotinamide observed in ex-situ perfused compared to native livers. Indeed, this finding suggests that NAD biosynthesis was triggered in liver cells likely as a feed-back mechanism secondary to the activation of NAD^+^-consuming enzymes Sirtuins. This latter biological event represents a crucial step in the induction of liver cell regeneration and response to stress^52,53^.

In line with our previous research in ex vivo lung perfusion^14,15^, these data shows that IR injury occur during NMP and that it is associated with specific biological events that must be investigated independently from transplant procedure. The present platform enables the evaluation of these events and offers some insights that could be useful in clinical NMP practice. In fact, although prolonged reperfusion of the liver has been already successfully achieved^5–7^, a systematic description of the impact of the procedure on hepatic homeostasis was still lacking.

From this point of view, the use of uninjured livers could represent a limitation in the translational value of our study. However, this is an essential prerequisite to any further research development, as it allows the identification of the changes specifically related to the machine perfusion procedure, while avoiding the wide range of variables related to organs with pre-existing damage. Consequently, although the absence of the transplantation procedure could be seen as a possible further limitation, the in-vivo reperfusion is not the aim of our investigations. Indeed, our results would represent a helpful starting point for the very many conditions of liver injury encountered in transplantation settings or for the exploitation of preclinical NMP as a liver research platform. Interestingly, we have showed that by integrating different biological analyses into a reproducible preclinical NMP model a broad description of IRI events is feasible. It should be noted that, while we have demonstrated that a resolution phase of IRI could be triggered during prolonged NMP, a proper liver regeneration is far from being achieved due to the actual intrinsic mechanisms of isolated liver perfusion.

In conclusion, this study demonstrates the possibility to successfully achieve a 12-h NMP in rat liver organs. At a bio-molecular level, no signs of oxidative stress, massive cell death, or tissue damage were detected in livers exposed to prolonged perfusion. Rather, enhanced production of factors involved in acute phase response and cell survival suggest activation of endogenous protective mechanisms. Overall, the metabolic and bio-molecular scenario observed in livers subjected to prolonged perfusion depicts a viable liver that was able to adapt its biological machinery to the different conditions experienced during the procedure. Our hypothesis is that during early NMP, adaptation responses are triggered within liver cells to overcome IRI, while prolongation of NMP leads to activation of repair and synthesis mechanisms. However, additional steps are needed to adequately support these events, otherwise the early beneficial effects of NMP could be rapidly lost over the prolonged ex-situ perfusion time course. For instance, since perfused livers showed extensive metabolic changes that could impact prolonged perfusion, our findings should push to further considerations on the perfusion fluid composition in the prolonged reperfusions. Despite the intrinsic limitation of a rodent NMP model, the present 12 h-NMP model could help future studies to better investigate the effects exerted by different stimuli and interventions on isolated rat livers, such as gene treatment, defatting, and cellular therapies^16^.

## Methods

### Animals and study design

All experimental procedures, approved by the Italian Ministry of Health (number 456/2021), were performed at the Center for Preclinical Research, Fondazione IRCCS Ca’ Granda Ospedale Maggiore Policlinico, Milan (Italy).

Adult Sprague–Dawley male rats weighing 354 ± 55 g (Envigo RMS. S.R.L, Udine, Italy) were housed in single ventilated cages systems (Tecniplast S.p.A., Varese, Italy) at 22 ± 1 °C, 55 ± 5% humidity, on a 12 h dark/light cycle, and were allowed to access food and water ad libitum.

Animals received humane care in compliance with the EU Directive 2010/63/EU and the Italian Legislative Decree 26/2014. Experiments were performed according to the 3R principles^24,25^ and the Planning Research and Experimental Procedures on Animals: Recommendations for Excellence (PREPARE) guidelines^54^. Sample size was calculated by a priori power analysis in compliance with the Animal Research: Reporting of In Vivo Experiments (ARRIVE) guidelines^55^ (details are provided in Supplementary material).

Rats were randomly assigned to the following study groups (n = 5 each) (Supplementary Fig. 9):

1. *Native (healthy control group)*—livers were simply procured immediately after induction of anesthesia;
2. *NMP4h*—livers were subjected to *in-situ* cold flushing with Celsior solution at 4 °C, with subsequent *ex-situ* perfusion for 4 h;
3. *NMP12h*—livers were subjected to the same procedures of NMP4h group, but the *ex-situ* perfusion was continued for 12 h.

### Anesthesia, surgery and in-situ perfusion

The drugs, reagents and instrumentation necessary to conduct this protocol were already described by our group^18^. Conversely, the anesthesia protocol was modified to reduce a possible toxic liver damage. Rats were anesthetized using isoflurane 5% (1 L/min O_2_ flow) for 10 min using a mouse/rat anesthetic induction chamber (Harvard Apparatus, Holliston, US). When unresponsive to pain, the rats were placed on the operating table and spontaneous breathing was ensured using a mask insufflating O_2_ and isoflurane 3%. After the induction of anesthesia, surgical procedure and organ procurement were carried on as previously described^17,18^.

### Ex-situ liver perfusion

#### Perfusion fluid preparation

A volume of 120 mL perfusion fluid was prepared. The composition of the perfusion fluid was slightly modified from our previous experiments^17^: 46.2 mL of Oxyglobin® (HbO2 Therapeutics; Souderton, PA, US), a non-cellular haemoglobin with 30 h half-life were added to 73.8 mL of mixed Williams Medium E, human albumin, glutamine, streptomycin/penicillin, insulin, N-acetylcysteine, sodium taurocholate (details on the reagents and concentrations are reported in Supplementary Table 2). Baseline perfusion fluid characteristics are described in Supplementary Table 1.

#### Normothermic machine perfusion setup

The NMP circuit was derived from our already described NMP circuit^18,56^. However, to reduce Meta-Oxyglobin formation and to keep the volume of perfusate constant, the circuit was modified as shown in Fig. 5 (details of the components of the circuit are provided in Supplementary Table 3). In particular, a reinfusion and a suction line were added to the circuit, and each line was connected to an infusion pump. The two pumps were set to maintain a constant 100 mL of perfusate volume (a volume corresponding to 20 mL of the initial 120 mL was removed from the returning fluid during the first 5 min of perfusion, namely wash-out) with a 20 mL/h perfusate substitution. Membrane lung oxygenation was achieved with a gas mixture of 50% O_2_-5% CO_2_ at a flow of 200 mL/min.

#### Ex-situ liver perfusion protocol

We used the standard NMP protocol implemented in our laboratory^18^. Exclusively the NMP duration was modified: “rewarming” phase lasted 40 min, and “normothermic” phase 4 or 12 h.

During the rewarming phase, the flow rate and temperature of the liver increased every 5 min until reaching the target flow (30 mL/min, max portal pressure 9 mmHg) and target temperature of the graft (37 °C). During the perfusion, hemodynamic parameters (portal flow and pressure) were monitored.

### Perfusate sample collection and analysis

Perfusate samples were hourly collected from the beginning of fluid re-circulation to the end of perfusion. Pre-liver and post-liver perfusates were used to assess acid–base balance, electrolytes, and metabolites using a gas analyzer (ABL 800 Flex; Radiometer, Copenhagen, Denmark).

To perform bio-molecular analyses, perfusate samples were processed following the procedure previously described^15,18,57^ to remove Oxyglobin (Supplementary methods). Bio-molecular evaluation involved the assessment of the concentration of biomarkers related to cytolysis, hepatocyte injury, extracellular matrix and glycocalyx turnover, liver acute phase response, liver sinusoidal endothelial cells (LSEC) activation, and oxidative damage. The release of flavin mononucleotide (FMN) was likewise assessed as previously described^58^. Materials and methods are detailed in the online Supplementary methods.

### Bile analysis

The 1.5 mL polypropylene tubes prefilled with 0.2 g of vaseline oil were used to collect bile and were changed hourly and stocked. After being weighted, the net weight was used to estimate bile production. Then, 100 µL were withdrawn and analyzed with the gas analyzer.

### Tissue sample collection and analysis

At the end of NMP, the liver graft was disconnected from the perfusion system and weighted. The right median lobe was resected to obtain different tissue biopsies that were subsequently blinded analyzed. Snap-frozen biopsies were used to determine adenosine triphosphate (ATP)^57^, nicotinamide adenine dinucleotide (NAD^+^)/NADH content, and malondialdehyde (MDA) while formalin-fixed samples were used to perform various stains, including the assessment of tissue integrity, glycogen content and Ki67%. In addition, snap-frozen tissue samples were used to perform ^1^H nuclear magnetic resonance (NMR) spectroscopy-based metabolomics, quantifying a total of 56 unique metabolites (Supplementary methods).

### Statistical analysis

Data are presented as mean ± standard deviation (SD) or standard error of the mean (SEM). Uptake ratio, oxygen consumption (VO_2_) and delivery (DO_2_) were calculated as described in Supplementary Material. Results of perfusate assessments were analyzed using two-way repeated measures analysis of variance (ANOVA). Data were rank-transformed if not normally distributed. T-test or one-way ANOVA followed by Tukey’s multiple comparison test was used to investigate differences in perfusate mediators and markers. Pearson’s correlation coefficients were calculated to investigate potential associations between variables. Univariate statistical analysis was performed using SigmaPlot 11.0 software (Systat Software Inc, San Jose, CA) and Prism GraphPad 9.5.1 (GraphPad Software, Boston, MA). Statistical analyses on metabolite data (Fig. 4) were performed with the MetaboAnalyst 5.0 web server (https://www.metaboanalyst.ca/MetaboAnalyst/ModuleView.xhtml). Briefly, data were normalised for dilution effects by the probabilistic quotient normalisation (PQN) approach on a reference sample. Heatmap was produced using non-parametric one-way ANOVA and unsupervised clustering was achieved by the Ward clustering algorithm employing the Euclidean distance measure. Autoscaled and normalised metabolite concentration values are shown. while metabolomics data was analyzed and visualized with MetaboAnalyst 5.0^59^. A probability value of < 0.05 was considered significant. Additional information is reported in the Supplementary methods.

### Supplementary Information

Supplementary Information.

## Data Availability

Data will be available under request at Daniele Dondossola (dondossola.daniele@gmail.com).

## Abbreviations

A2M: Alpha 2 macroglobulin
AGP: Alpha-1 acid glycoprotein
ARG1: Arginase 1
ATP: Adenosine triphosphate
DO_2_: Oxygen delivery
FMN: Flavin mononucleotide
GSSG: Glutathione disulfide—oxidized form of glutathione
HOPE: Hypothermic oxygenated machine perfusion
hRBC: Human red blood cells
IRI: Ischemia-reperfusion injury
LSEC: Liver sinusoidal endothelial cells
LT: Liver transplantation
MDA: Malondialdehyde
MP: Machine perfusion
NAD+: Nicotinamide adenine dinucleotide
NMP: Normothermic machine perfusion
NMR: ^1^H nuclear magnetic resonance
OxC: Oxygen carrier
RBC: Red blood cells
SD: Standard deviation
SEM: Standard error of the mean
VO_2_: Oxygen consumption
5′-NT/CD73: 5′-Nucleotidase
8-OHdG: 8-Hydroxydeoxyguanosine
