# Supplementary material for: Twelve-hour normothermic liver perfusion in a rat model: characterization of the changes in the ex-situ bio-molecular phenotype and metabolism
Source: Sci Rep. 2024 Mar 13;14:6040. doi: 10.1038/s41598-024-56433-3 (PMC10933381; doi:10.1038/s41598-024-56433-3)
Supplement: Supplementary file 1 — Supplementary Information. [file 41598_2024_56433_MOESM1_ESM.docx]

**Supplementary Material Index**

[1. Supplementary Methods 3](#_Toc142384243)

[1.1. Perfusate analyses 3](#_Toc142384244)

[1.1.1. Processing and analysis of perfusate samples 3](#_Toc142384245)

[1.1.2. Adenylate kinase (AK) and Caspase cleaved cytokeratin 18 (CK18) 3](#_Toc142384246)

[1.1.3. Flavin mononucleotide (FMN) 3](#_Toc142384247)

[1.1.4. Luminex xMAP® Technology immunofluorescent assays 3](#_Toc142384248)

[1.1.5. Assessment of 8-hydroxy-2'-deoxyguanosine” (8-OHdG) 4](#_Toc142384249)

[1.1.6. Glycosaminoglycan (GAG) determination 4](#_Toc142384250)

[1.1.7. Nicotinamide adenine dinucleotide + H^+^ (NADH) measurement 4](#_Toc142384251)

[1.2. Tissue analysis 4](#_Toc142384252)

[1.2.1. Nicotinamide adenine dinucleotide (NAD)^+^/NADH measurement 4](#_Toc142384253)

[1.2.2. Malondialdehyde (MDA) measurement 4](#_Toc142384254)

[1.2.3. ATP content evaluation 5](#_Toc142384255)

[1.2.4. Nuclear magnetic resonance (NMR) spectroscopy-based metabolomics 5](#_Toc142384256)

[1.3. Sample size calculation 6](#_Toc142384257)

[1.4. VO_2_ and DO_2_ 6](#_Toc142384258)

[2. Supplementary results 8](#_Toc142384259)

[3. Supplementary Tables 9](#_Toc142384260)

[3.1. Supplementary table 1. Composition of the perfusion fluid supplemented with acellular Oxygen carrier (Oxyglobin®) 9](#_Toc142384261)

[3.2. Supplementary table 2. Perfusion fluid characteristics before graft connection to the NMP circuit and after 4 h and 12 h of perfusion. 10](#_Toc142384262)

[4. Supplementary Figures 11](#_Toc142384263)

[4.1. Supplementary figure 1. Schematic representation of experimental workflow and sample collection. 11](#_Toc142384264)

[4.2. Supplementary figure 2. Portal vein flow and resistance monitoring over NMP. 12](#_Toc142384265)

[4.3. Supplementary figure 3. Perfusate glucose concentration over the NMP procedure. 13](#_Toc142384266)

[4.4. Supplementary figure 4. Lab test of perfusate samples 14](#_Toc142384267)

[4.5. Supplementary figure 5. Perfusate FMN over the NMP procedure. 15](#_Toc142384268)

[4.6. Supplementary figure 6. Histopathology of the liver tissue at the end of NMP compared to Native livers. 16](#_Toc142384269)

[4.7. Supplementary figure 7. Release of Glycosaminoglycan (GAG) fragments over the 12 h-NMP procedure. 17](#_Toc142384270)

[4.8. Supplementary figure 8. NADH release in the perfusate over the 12 h-NMP procedure. 18](#_Toc142384271)

[4.9. Supplementary figure 9. Liver tissue metabolomics analysis. 19](#_Toc142384272)

[5. References 20](#_Toc142384273)

# Supplementary Methods

## Perfusate analyses

### Processing and analysis of perfusate samples

Perfusate samples were centrifugated at 2000 rpm for 10 min at 4°C (Haereus Multifuge X3R, Thermo Fisher Scientific Inc., Cambridge, UK). Supernatants were collected and purified with Amicon Ultra 100K centrifugal filter devices (Millipore Corporation) to avoid colorimetric interferences possibly given by Oxyglobin®. Briefly, 500 µL of each sample were loaded onto the columns and centrifuged at 13500 rpm for 15 min (Haereus Multifuge X3R). Next, Oxyglobin-free eluates were collected and stored at -80°C for subsequent bio-molecular analysis.

### Adenylate kinase (AK) and Caspase cleaved cytokeratin 18 (CK18)

Perfusate Adenylate kinase (AK) and Caspase cleaved cytokeratin 18 (CK18) were assessed as indexes of cell death by means of a bioluminescent assay (ToxiLight BioAssay, Lonza, Basel, Switzerland) and ELISA assay (Thermo Fisher Scientific, Waltham, MA, USA). Luminescence detection and absorbance reading were performed using a multi-mode microplate reader (Synergy HTX, Biotek U.S, Winooski, VT, USA) and a luminometer (Glomax Luminometer; Promega, Madison, WI, USA), respectively.

### Flavin mononucleotide (FMN)

The FMN concentration was determined by fluorescence spectroscopy. Perfusate samples were dispensed in triplicate in black microplates. Fluorescence readings were performed using a multi-mode microplate reader (Synergy HTX). A monochrome light with excitation wavelength of 460/40 nm was used, while fluorescence emission was revealed with 100% gain at 528/20 nm.

### Luminex xMAP® Technology immunofluorescent assays

Perfusates were diluted 1:10 with 1X PBS solution (Sigma-Aldrich, Saint Louis, USA) and then added V/V to Hemoglobind (Biotech Support group, Monmouth Junction, NJ, USA). After 10 min-vigorous mixing at room temperature (RT), samples were subjected to 4 min-centrifugation at 10,000 rpm (Haereus Multifuge X3R). Supernatants were recovered and stored at -80 °C. Oxyglobin-free samples were used to assess selected biomarkers of liver injury, liver response to stress stimulus, and liver sinusoidal endothelial cells (LSEC) activation. Analyte concentration was measured using custom-designed immunoassays based on Luminex xMAP® Technology (Milliplex multiplex assays, Merck KGaA). Fluorescence signals were detected on a Luminex 200 platform (Austin, CA, USA).

### Assessment of 8-hydroxy-2'-deoxyguanosine” (8-OHdG)

Generation of the oxidized form of 2’-deoxyguanosine was determined to assess the occurrence of oxidative damage to DNA (DNA damage competitive ELISA, Thermo Fisher Scientific). Absorbance reading was performed at 450 nm using a multi-mode microplate reader (Synergy HTX).

### Glycosaminoglycan (GAG) determination

Glycosaminoglycan (GAG) released in the perfusate were measured using a commercially available ELISA assay (Cusabio Technology LLC, Wuhan, Hubei Province, China). Absorbance reading was performed at 450 nm using a multi-mode microplate reader (Synergy HTX).

### Nicotinamide adenine dinucleotide + H^+^ (NADH) measurement

Perfusate NADH was assessed by measuring fluorescence signals according to a validated method (1). Briefly, perfusate samples were exposed to 340 nm excitation wavelength and 445 nm emission peak using a multi-mode microplate reader (Synergy HTX). A standard curve ranging from 1 to 500 μM NADH was used to determine the concentration of NADH in each sample.

## Tissue analysis

### Nicotinamide adenine dinucleotide (NAD)^+^/NADH measurement

Oxidized NAD and its reduced form NADH were assessed in hepatic tissue using a commercially available kit (Sigma-Aldrich). Briefly, snap-frozen liver biopsies were immerged in an appropriate volume of extraction buffer and then homogenized. Thereafter, tissue extracts were deproteinized using 10kDa cut-off spin filters (Millipore - Amicon Centrifugal Filter Unit, Merck&Co, Kenilworth, NJ, USA).

Detection of total NADH and NAD (NAD_total_) was carried out in parallel. Selective measurement of NADH was performed after destroying NAD by heating samples at 60 °C for 30 min. Both untreated and NAD-free tissue extracts were dispensed in duplicate in 96-well plates, added with NAD Cycling Enzyme and NADH developer, and incubated for 2 h at RT. After stopping the reaction, absorbance readings were performed at 450 nm using a multi-mode microplate reader (Synergy HTX). To calculate NADH and NAD_total_ concentrations, absorbance of unknown samples was compared to those of standard samples.

### Malondialdehyde (MDA) measurement

Malondialdehyde, a marker for lipid peroxidation was measured in snap-frozen liver biopsies using a colorimetric assay kit (Sigma-Aldrich). Briefly, tissue specimens were homogenized on ice using the specific buffer provided by the kit. Liver homogenates were then subjected to centrifugation at 13.000g for 10 min (Haereus Multifuge X3R). The collected supernatants were added with thiobarbituric acid to form a colorimetric product. Absorbance readings were performed at 532 nm using a multi-mode microplate reader (Synergy HTX).

### ATP content evaluation

Tissue ATP was evaluated as previously described (2). Briefly, Snap-frozen liver biopsies were homogenized in 2.5% trichloroacetic acid (Sigma-Aldrich) and then subjected to 10 min centrifugation at maximum speed at 4 °C (Haereus Multifuge X3R). Supernatants were diluted 1:30 using 0.1 M Tris-acetate pH 7.75 (Sigma-Aldrich). ATP content was then determined using a luciferase-based assay (Enliten ATP Assay System, Promega). Bioluminescent signals were detected with a luminometer (Glomax Luminometer, Promega). ATP concentration was calculated using a standard curve ranging from 10^-11^ to 10^-5^ M (rATP 10 mM, Promega). Results were expressed as concentration/wet tissue weight.

### Nuclear magnetic resonance (NMR) spectroscopy-based metabolomics

NMR spectroscopy-based metabolomic analysis was performed in liver tissue homogenates to selectively highlight key metabolic pathways.

Cryogenically pulverized deep-frozen liver biopsies (Covaris cryoPREP CP02, Woburn, MA, USA) were transferred to Covaris system-compatible glass tubes, suspended in 300 μL LC-MS purity grade methanol (Sigma Aldrich Chemie, Taufkirchen, Germany), added with 1000 μL tert-butylmethyl ether (Sigma Aldrich Chemie), and then subjected to ultra-sonication-based metabolite extraction in liquid state (Covaris E220 Evolution, Woburn, MA, USA). After ultra-sonication, 250 μL of molecular biology purity grade water were added and the tubes were centrifuged for phase separation (12’000 g x 20 min). The aqueous layers were collected, transferred to 1.5 mL tubes and evaporated overnight. The obtained dried pellets were re-suspended in deuterated phosphate buffer solution (pH = 7.4; 1M K_2_HPO_4_; 10 mM NaN_3_; all reagents were purchased from Sigma Aldrich Chemie) containing 1 mM internal NMR spectroscopy reference standard 3-(trimethylsilyl) propionic-2,2,3,3-d_4_ acid sodium salt (TSP) (Sigma Aldrich Chemie). Then, the samples were centrifuged (30’000 g x 30 min) to remove any undissolved particles and the supernatants were filled into 3mm Bruker SampleJet-compatible NMR spectroscopy sample tubes (Bruker BioSpin, Ettlingen, Germany).

NMR spectra were acquired on a Bruker Avance III HD 14.10 Tesla spectrometer (600 MHz for 1H, Bruker BioSpin) with a 1.7 mm triple-resonance RT probe (Bruker BioSpin), using Carr-Purcell-Meiboom-Gill (CPMG) pulse sequences lasting from 1 h to 4 h depending on the amount of liver powder originally loaded. NMR signals were pre-processed with Bruker TopSpin 3.6.1 software (Bruker Biospin). Targeting and semi-quantitative analysis were performed using ChenomX NMR suite 8.5 software (Chenomx Inc., Alberta, Canada).

## Sample size calculation

An “a priori” power analysis was carried out to estimate the minimum number of animals needed to reliably detect the expected effect size, in agreement with the principle of Reduction (3,4).

Given the well-known negative effects of reperfusion on mitochondrial oxidative phosphorylation, ATP liver content was used as reference variable to explore differences between the study groups. More specifically, we assumed an expected effect size between the NMP12 and the Native groups of 70%, with a standard deviation of 30%. Details on sample size calculation are listed below:

- number of groups: 3 (Native, NMP4h, NMP12h);
- statistical test: one-way or two-way ANOVA;
- expected effect size: 70%;
- expected standard deviation: 30%;
- desired power: 0.80;
- alpha error: 0.05.

The analysis provided a minimum number of 5 livers for each experimental group. The final number needed to perform the whole study was therefore 15.

## VO_2_ and DO_2_

A modified Fick equation was used to calculate oxygen delivery (DO_2_) and oxygen consumption (V̇O_2_) in our experiments. Cardiac output was intended as the pump flow; pre-liver perfusate samples were intended as O_2_ enriched perfusate (arterial blood of the Fick equation), whereas post-liver perfusate samples, collected directly from the inferior cava vein, were used for the calculation of venous oxygen content.

[1,34 x Hb (g/dL) _PRE-LIVER_ x HbO_2_(%)] + 0,003 x P _PRE-LIVER_O_2_(mmHg) =

**Oxygen content of the pre-liver perfusate (C_PRE_O_2_)**

[1,34 x Hb (g/dL) _POST-LIVER_ x HbO_2_(%)] + 0,003 x P _POST-LIVER_O_2_ (mmHg) =

**Oxygen content of the post-liver perfusate (C_POST_O_2_)**

Oxygen delivery was measured as follows:

**DO_2_** = C _PRE_O_2_ x Pump flow (mL/min) / 100

Similarly, **V̇O_2_** = (C _PRE_O_2_ – C_POST_O_2_ ) x Pump flow (mL/min) / 100

# Supplementary results

Potassium increased during the NMP4h with a potassium uptake ratio of -0.24±0.19 and a final value of 4.9±0.9 mEq/L, while in NMP12h it increased during first 6h of perfusion and then remained stable with a potassium uptake ratio -0.39±0.08 and a final value of 4.7±0.3 mEq/L (p=0.395).

Bicarbonate level was stable around 9±2 mmol/L in NMP4h and 10±1 mmol/L in NMP12h with a base excess of -19±3 mmol/L in NMP4h and -18±2 mmol/L NMP12h with a resulting pH of 6.9±0.2 NMP4h and 7.0±0.1 NMP12h

# Supplementary Tables

## Supplementary table 1. Perfusion fluid characteristics before graft connection to the NMP circuit and after 4 h and 12 h of perfusion.

All the parameters were measured both in pre- and post-liver perfusate samples. Data are presented as mean±SD. Abbreviations: BE, base excess.

|  | **NMP4h** | | | **NMP12h** | | | | |
| --- | --- | --- | --- | --- | --- | --- | --- | --- |
|  | **Priming** | **4h Pre** | **4h Post** | **Priming** | **4h Pre** | **4h Post** | **12h Pre** | **12h Post** |
| **pH** | 7.00±0.08 | 7.03±0.03 | 6.99±0.03 | 7.02±0.04 | 7.04±0.02 | 6.99±0.02 | 7.05±0.04 | 7.03±0.05 |
| **pCO_2_, mmHg** | 39±11 | 38±2 | 46±2 | 38±5 | 40±2 | 47±2 | 39±1 | 44±2 |
| **pO_2_, mmHg** | 377±64 | 293±25 | 53±9 | 386±21 | 261±23 | 39±12 | 259±14 | 58±10 |
| **Hb, g/dL** | 5.2±0.1 | 5.5±0.1 | 5.5±0.1 | 5.2±0.2 | 5.5±0.1 | 5.5±0.1 | 5.6±0.1 | 5.6±0.1 |
| **Met-Oxyglobin, %** | 14±6 | 29±3 | 28±3 | 15±3 | 31±4 | 30±4 | 35±4 | 34±4 |
| **K^+^, mEq/L** | 3. 6±0.1 | 4.4±0.2 | 4.5±0.2 | 3.4±0.1 | 4.2±0.3 | 4.2±0.2 | 4.8±0.2 | 4.8±2 |
| **Na^+^, mEq/L** | 149±2 | 160±2 | 161±2 | 146±3 | 158±4 | 159±4 | 158±5 | 160±5 |
| **Glucose, mg/dL** | 73±5 | 264±31 | 262±32 | 74±4 | 261±43 | 261±38 | 148±22 | 149±21 |
| **Lactate, mmol/L** | 3.9±0.7 | 2.5±0.8 | 2.5±0.8 | 3.8±0.2 | 2.4±0.4 | 2.4±0.4 | 1.1±0.4 | 1.1±0.4 |
| **BE** | -20±0.2 | -18.8±1.0 | -18.5±0.8 | -19.7±0.5 | -18.4±0.8 | -18.2±0.6 | -18.0±1.3 | -17.7±1.6 |
| **HCO_3_ ^-^, mmol/L** | 8.9±1.0 | 9.8±0.7 | 10.6±0.5 | 9.2±0.5 | 10.2±0.6 | 10.9±0.4 | 10.4±0.8 | 11.0±1.0 |

## Supplementary table 2. Composition of the perfusion fluid supplemented with acellular Oxygen carrier (Oxyglobin®)

|  | **mL** | **Concentration** |
| --- | --- | --- |
| **William's Medium E**  *Gibco; Thermo Fisher Scientific, Waltham, MA, US* | 44.3 | - |
| **Oxyglobin®** (13 g/dL hemoglobin)  *HbO2 Therapeutics; Souderton, PA, US* | 46.2 | 5 g/dL |
| **Human Albumin** (200 g/L)  *Grifols; Barcelona, Spain* | 24 | 4 g/dL |
| **Pen-Strep** (10000 U/mL – 10 mg/mL)  *Gibco; Thermo Fisher Scientific, Waltham, MA, US* | 1.2 | 100 U/mL – 0.1 mg/mL |
| **L-Glutamine** (200 mM)  *Gibco; Thermo Fisher Scientific, Waltham, MA, US* | 1.2 | 0.292 mg/mL |
| **Insulin** (100 UI/mL)  *Humalog, Eli Lilly Nederland B.V.; Utrecht, Netherlands* | 2.4 | 2 U/mL |
| **N-acetylcysteine** (100 mg/mL)  *Zambon S.p.A.; Bresso, Italy* | 0.6 | 0.5 mg/mL |
| **Sodium taurocholate** (243 mg/mL)  *Sigma-Aldrich; Merck KGaA, Darmstadt, Germany* | 0.1 | 0.20 mg/mL |
| **Total Volume** | 120 |  |

## Supplementary table 3. Details of the instruments used and of the components of the normothermic machine perfusion circuit

| **Roller pump** | ISM834A; Ismatec SA, Glattburg, Switzerland |
| --- | --- |
| **Heat exchanger** | EcoLine 003 E100; Lauda Dr. R. Wobser Gmbh & Co. Kg, Lauda-Königshofen, Germany |
| **Blood gas analyzer** | ABL 825 Flex; Radiometer, Copenhagen, Denmark |
| **Data acquisition system** | PowerLab 16/35; ADInstruments, Dunedin, New Zealand |
| **Data acquisition software** | Labchart 8; ADInstruments, Dunedin, New Zealand |
| **Temperature Probe** | MLT1401; ADInstruments, Dunedin, New Zealand |
| **Double-headed surgical microscope** | OPMI 1- Zeiss West Germany, Oberkochen, Germany |
| **Circulating tube** | Volumat Line VL ST00; Fresenius Kabi, Ba​​d Homburg, Germany |
| **Membrane oxygenator** | Micro-1 Rat Oxygenator, Dongguan Kewei Medical Instrument Co., Ltd., Guangdong, China |
| **Infusion Pump** | Volumat MC Agilia; Fresenius Kabi, Ba​​d Homburg, Germany |
| **Pressure transducer** | TruWave; Edwards Lifesciences, Irvine, California, U.S. |
| **Parafilm** | Pechiney Plastic Packaging, Menasha, Wisconsin, U.S. |
| **Glass reservoir** | Hugo Sachs Elektronik - Harvard Apparatus GmbH, March-Hugstetten Germany |
| **Bubble trap and heating coil** | Hugo Sachs Elektronik - Harvard Apparatus GmbH, March-Hugstetten Germany |
| **16 gauge cannula** | Introcan-W Certo, B. Braun, Melsungen, Germany |

# Supplementary Figures

## Supplementary figure 1. Portal vein flow and resistance monitoring over NMP.

Portal vein flow and resistance remained stable throughout the “normothermic perfusion phase”. Two-way RM ANOVA.


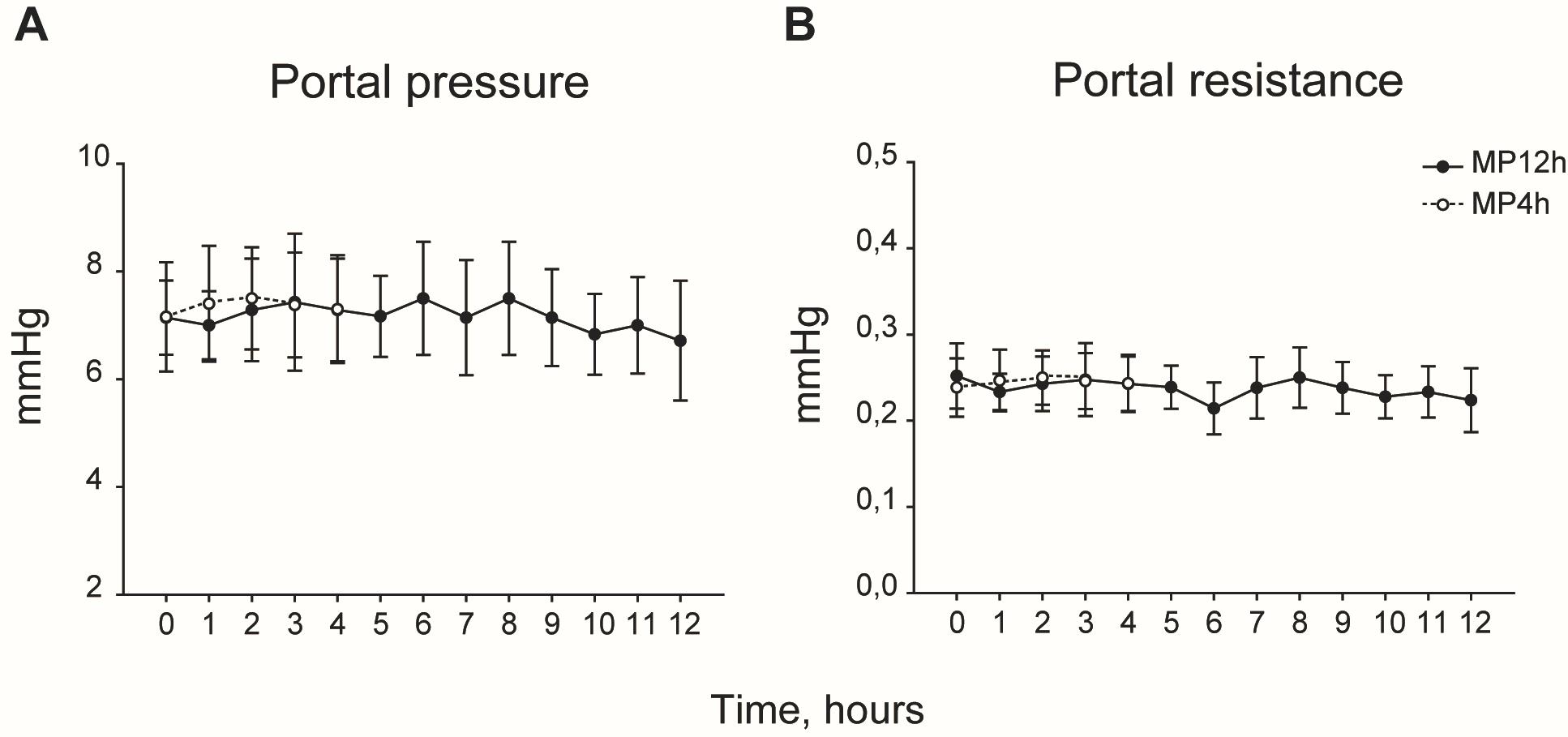


**NMP4h**

**NMP12h**

## Supplementary figure 2. Perfusate glucose concentration over the NMP procedure.

Glucose concentration decreased over NMP time (p=0.003 vs time; p=523 vs 4 h). Two-way repeated measures ANOVA, Tukey’s post hoc test.

## Supplementary figure 3. Lab tests of perfusate samples

Alanine-amino transferase (ALT), lactate dehydrogenase (LDH) and urea (BUN) content of the perfusate. Measures are expressed as total content per gram of liver (U/g for AST and LDH, mg/g for BUN). One-way repeated measures ANOVA, Tukey’s post hoc test; p-value<0.05: * vs 0 h; § vs 1 h; # vs 4 h.


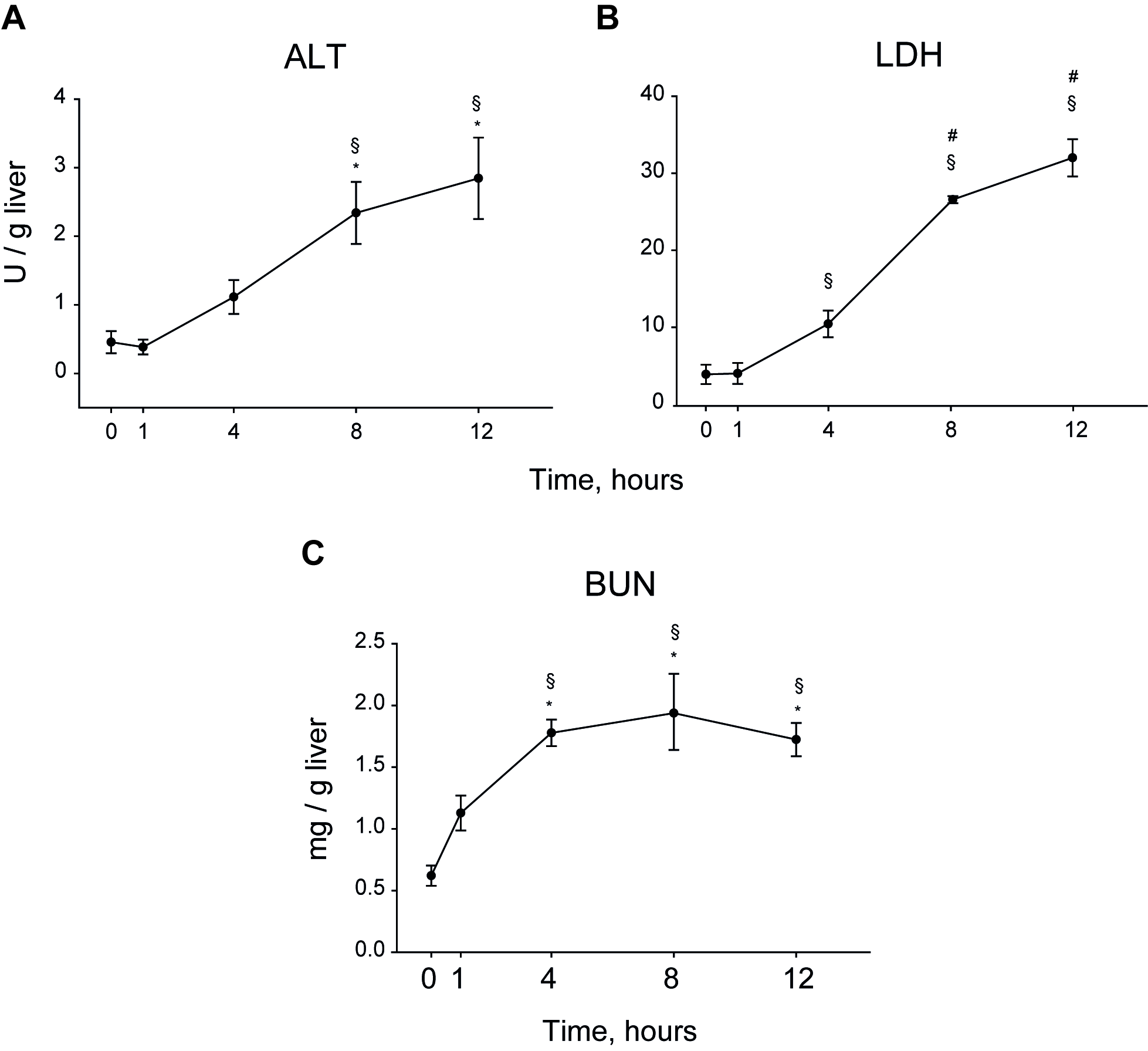


## Supplementary figure 4. Perfusate FMN over the NMP procedure.

One-way repeated measures ANOVA, Tukey’s post hoc test; vs Wash-out (W): * p<0,05; vs 1 h § p<0.05; vs 4 h # p<0.05.


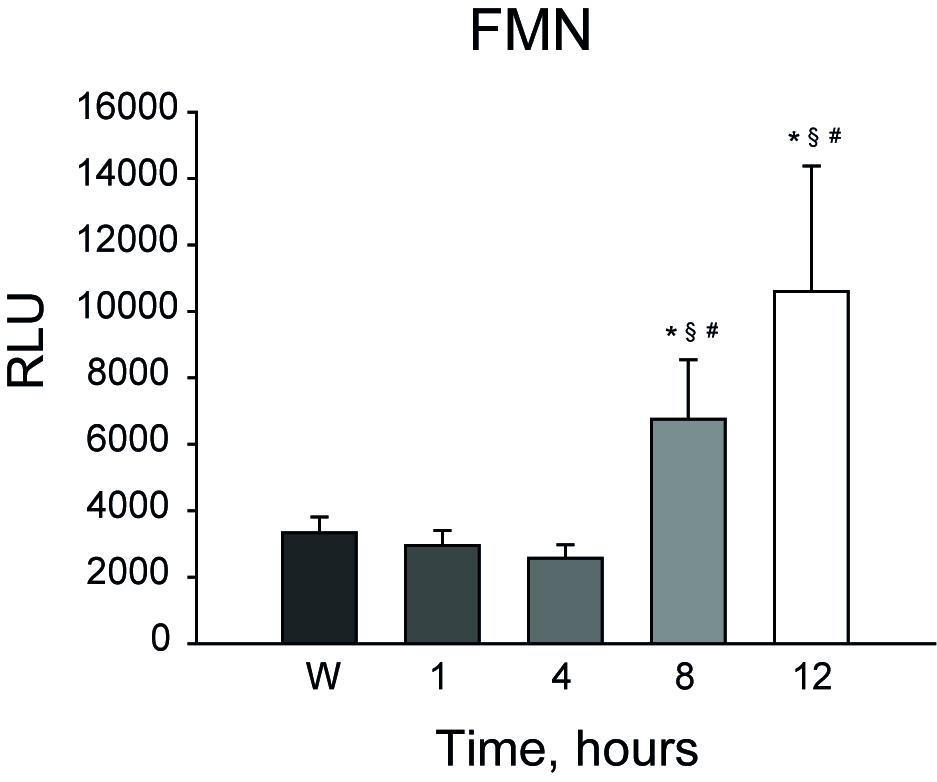


## Supplementary figure 5. PAS staining of the liver tissue at the end of 12 hours perfusion

Representative images of PAS staining (magnification 5x) of the tissue of all livers at the end of 12 hours NMP

**
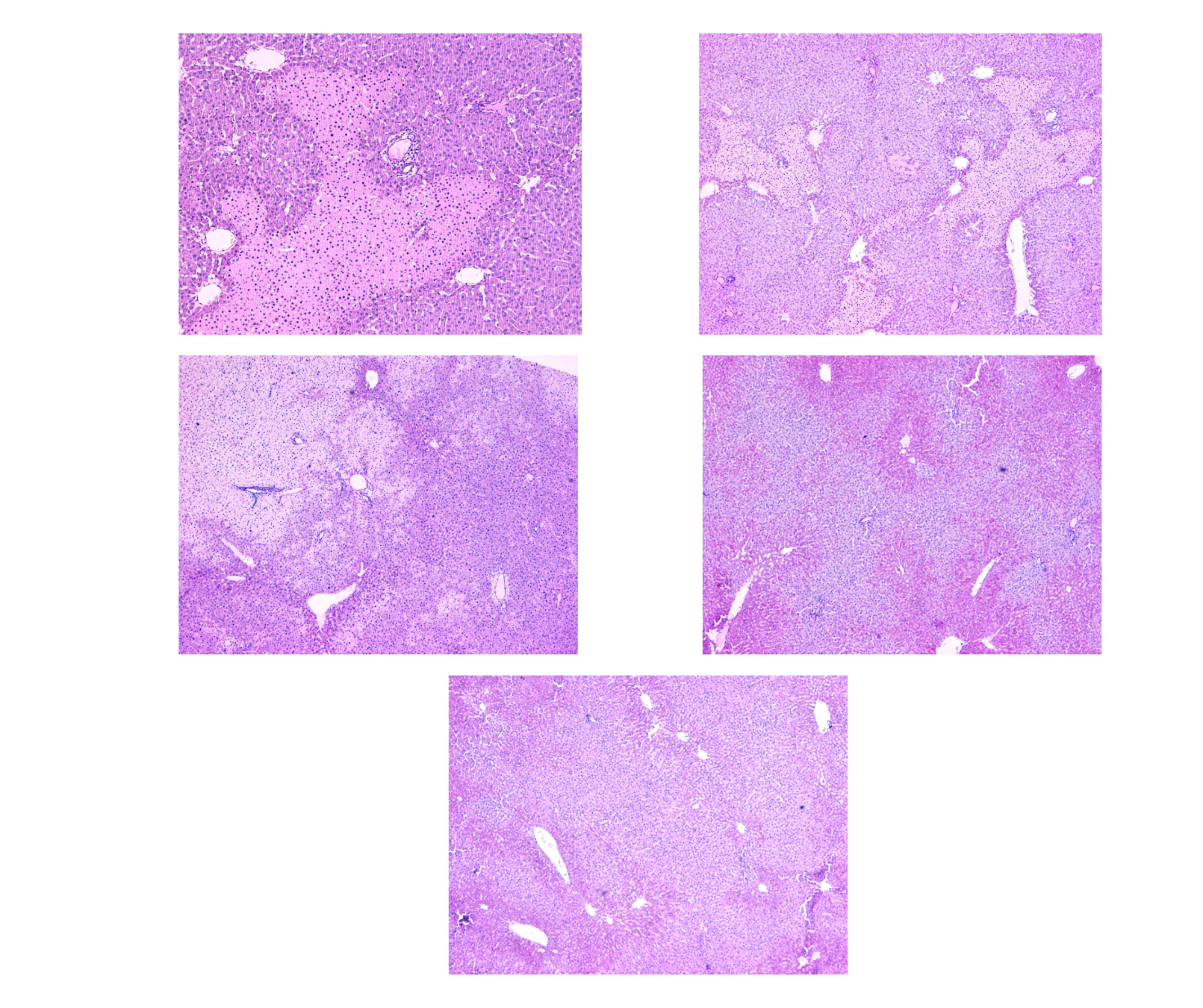
**

## Supplementary figure 6. Release of Glycosaminoglycan (GAG) fragments over the 12 h-NMP procedure.

GAG release was measured in perfusate samples collected during prolonged MP. One-way repetitive measures ANOVA, Tukey’s post hoc test; p values: ** p < 0.01 vs wash-out (WO).


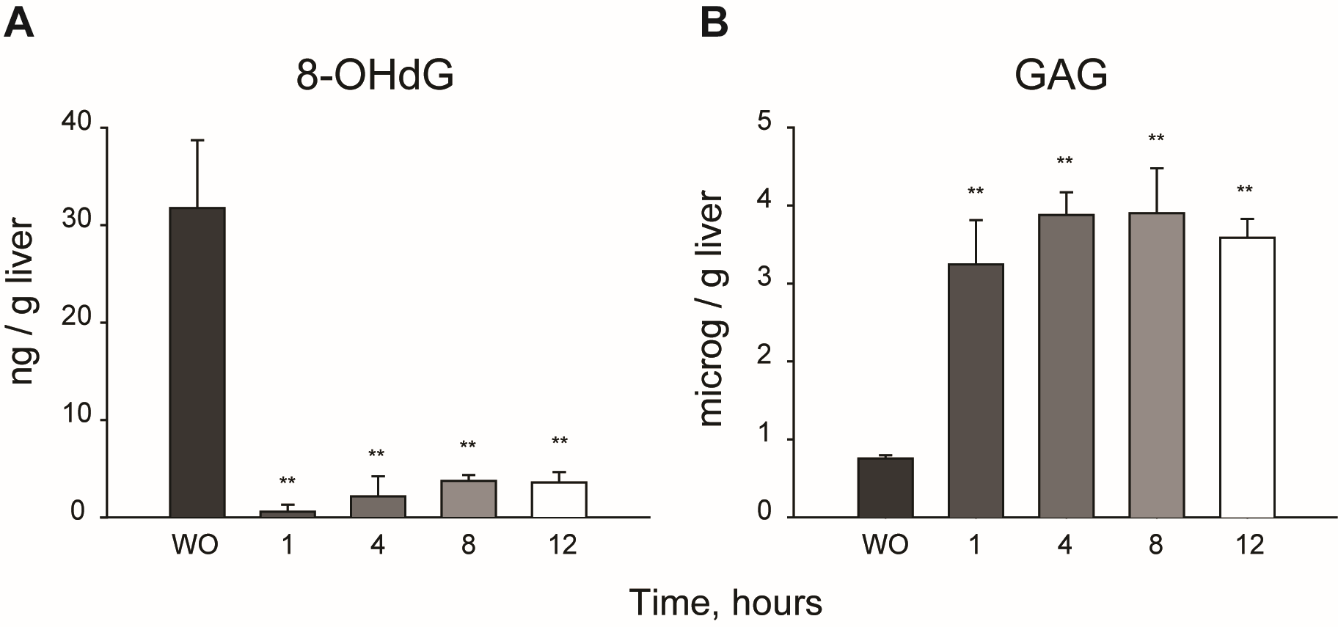


## Supplementary figure 7. NADH release in the perfusate over the 12 h-NMP procedure.

One-way repeated measures ANOVA, Tukey’s post hoc test; p value vs Wash-out (W) *** p<0.001; p value vs 1 h §§§ p<0.001; p value vs 4 h ### p<0.001; p value vs 8 h °°° p<0.001.

**
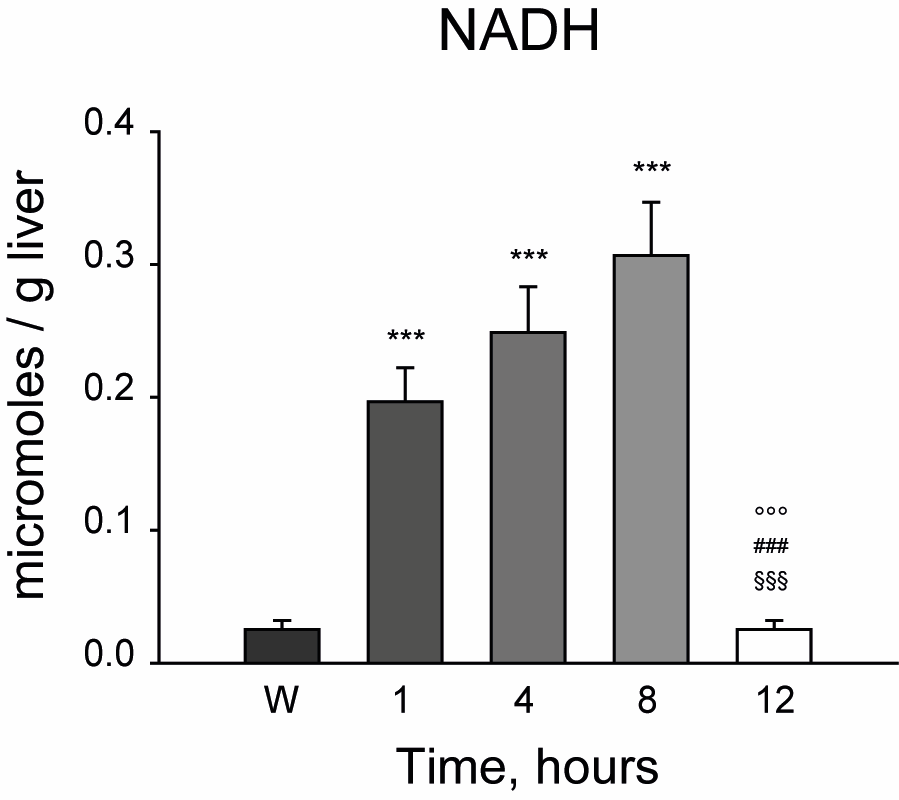
**

## Supplementary figure 8. Liver tissue metabolomics analysis.

A) Full averaged heatmap of quantified metabolite averaged concentrations, Ward clustering algorithm. B) Principal component analysis (PCA) scores plot illustrating general group separation based on metabolite concentration differences. C) Pattern hunter correlation analysis of glucose, illustrating positive and negative correlating metabolite concentration patterns within the dataset.


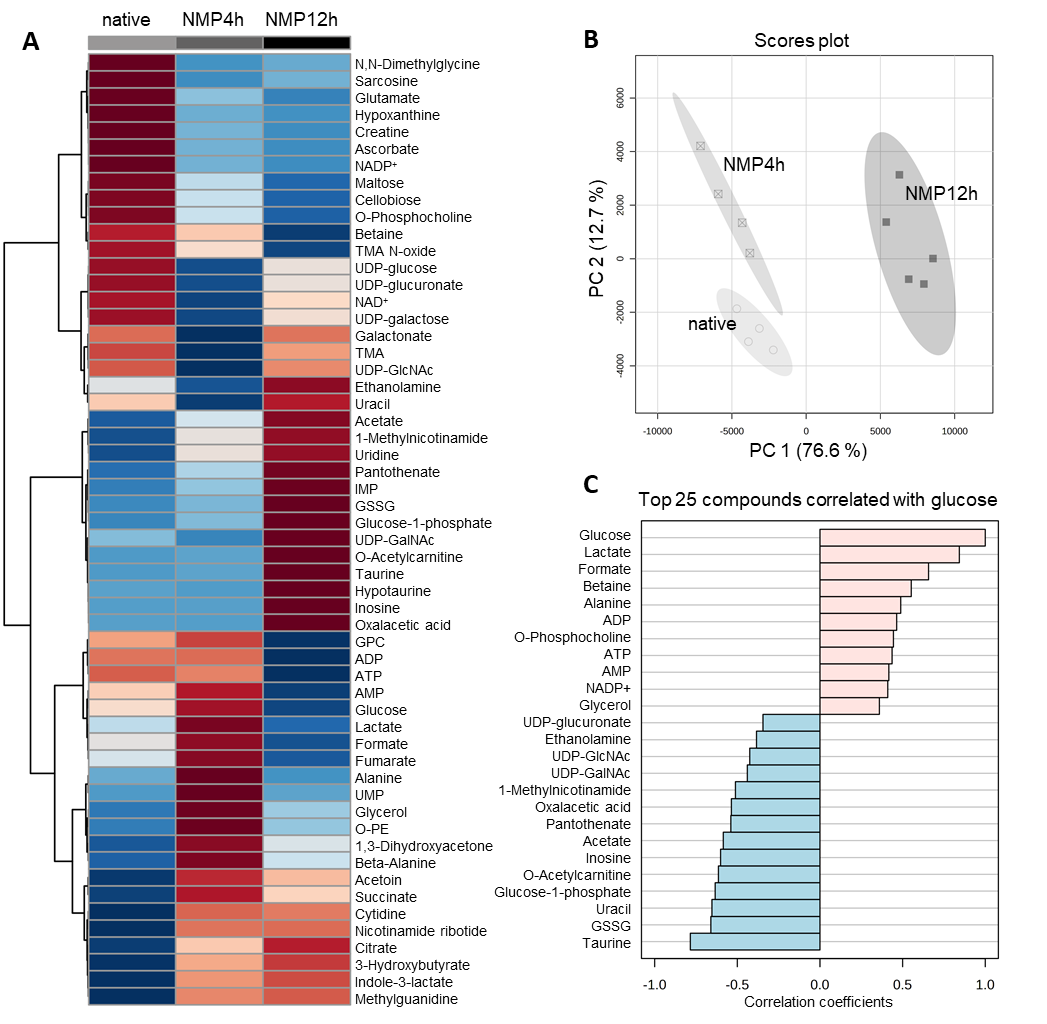


## Supplementary figure 9. Schematic representation of experimental workflow and sample collection.

After procurement, the liver grafts that underwent normothermic machine perfusion (NMP) were connected to the circuit. A 40 min rewarming phase was then applied. During the first 5 min of this phase, 20 mL of perfusate were collected and analyzed (*, washout). At 0 min, normothermia was reached with constant portal flow and pressure. Perfusate samples were collected hourly for blood gas analyses, while at 1 h, 4 h, 8 h, 12 h for biological evaluation (when feasible). At the end of NMP (2 h or 12 h duration), liver tissue was sampled for analyses.


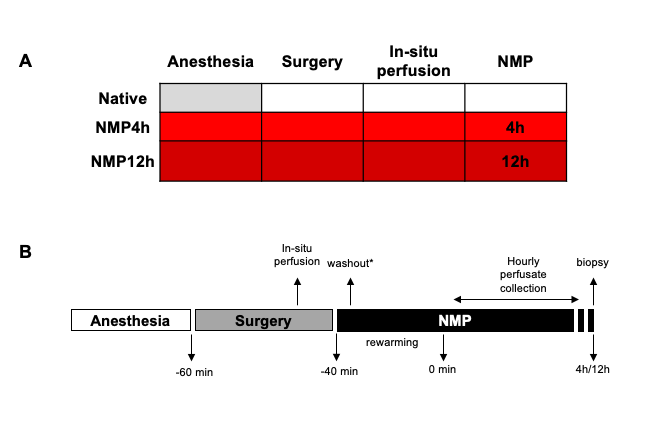


# References

1. Hast P. Determination of NADH Concentrations with the Synergy^TM^ 2 Multi-Detection Microplate Reader using Fluorescence or Absorbance. 2007.

2. Lonati C, Battistin M, Dondossola DE, Bassani GA, Brambilla D, Merighi R, et al. NDP-MSH treatment recovers marginal lungs during ex vivo lung perfusion (EVLP). Peptides. 2021;141(December 2020).

3. Balls M. It’s Time to Reconsider The Principles of Humane Experimental Technique. ATLA Altern to Lab Anim. 2020;48(1):40–6.

4. Russell WMS, Burch RL. The Principles of Humane Experimental Technique. Methuen Co, Ltd. 1959;
